# Implementation of the Randomized Embedded Multifactorial Adaptive Platform for COVID-19 (REMAP-COVID) trial in a US health system—lessons learned and recommendations

**DOI:** 10.1186/s13063-020-04997-6

**Published:** 2021-01-28

**Authors:** The UPMC REMAP-COVID Group, on behalf of the REMAP-CAP Investigators, David T. Huang, Bryan J. McVerry, Christopher Horvat, Peter W. Adams, Scott Berry, Meredith Buxton, Gilles Clermont, William Garrard, Timothy D. Girard, Ghady Haidar, Andrew J. King, Kelsey Linstrum, Salim Malakouti, Florian B. Mayr, Erin K. McCreary, Stephanie K. Montgomery, Christopher W. Seymour, Alexandra Weissman, Derek C. Angus

**Affiliations:** 1 University of Pittsburgh, 606B Scaife Hall, Pittsburgh, PA 15213 USA; 2 UPMC Health System, Northwest 628, Pittsburgh, PA 15261 USA

**Keywords:** Coronavirus, Clinical trial, Adaptive trial, Platform trial, SARS-CoV-2, COVID-19

## Abstract

**Background:**

The Randomized Embedded Multifactorial Adaptive Platform for COVID-19 (REMAP-COVID) trial is a global adaptive platform trial of hospitalized patients with COVID-19. We describe implementation at the first US site, the UPMC health system, and offer recommendations for implementation at other sites.

**Methods:**

To implement REMAP-COVID, we focused on six major areas: engaging leadership, trial embedment, remote consent and enrollment, regulatory compliance, modification of traditional trial management procedures, and alignment with other COVID-19 studies.

**Results:**

We recommend aligning institutional and trial goals and sharing a vision of REMAP-COVID implementation as groundwork for learning health system development. Embedment of trial procedures into routine care processes, existing institutional structures, and the electronic health record promotes efficiency and integration of clinical care and clinical research. Remote consent and enrollment can be facilitated by engaging bedside providers and leveraging institutional videoconferencing tools. Coordination with the central institutional review board will expedite the approval process. Protocol adherence, adverse event monitoring, and data collection and export can be facilitated by building electronic health record processes, though implementation can start using traditional clinical trial tools. Lastly, establishment of a centralized institutional process optimizes coordination of COVID-19 studies.

**Conclusions:**

Implementation of the REMAP-COVID trial within a large US healthcare system is feasible and facilitated by multidisciplinary collaboration. This investment establishes important groundwork for future learning health system endeavors.

**Trial registration:**

NCT02735707. Registered on 13 April 2016.

A Randomized Embedded Multifactorial Adaptive Platform (REMAP) trial combines features of adaptive platform and pragmatic point-of-care trials to simultaneously evaluate multiple treatment strategies and maximize trial conduct efficiency [1, 2].

REMAP-CAP is a global adaptive platform trial of patients with severe community-acquired pneumonia (CAP) admitted to the intensive care unit (ICU) that was launched in 2016 [3]. The trial design and rationale have been previously published [4]. Briefly, REMAP-CAP is governed by an International Trial Steering Committee (ITSC) and uses a core protocol that defines broad eligibility criteria, outcomes, and the statistical analysis plan. Multiple investigational treatments are layered within the core protocol as “domains”. Domains test investigational treatments with similar mechanisms of action, and multiple domains are simultaneously tested such that patients are randomized within each domain for which they are eligible. Testing of interactions between domains can be predefined or performed post hoc. As the number of domains increases, the likelihood a patient receives at least one investigational treatment increases accordingly. Randomization adapts as the trial evolves such that subjects are preferentially randomized to receive better performing arms based on interim analyses—termed “response adaptive randomization” [5]. Adaptations occur approximately monthly and use data from patients from all sites [4]. Domains are flexible such that additional investigational treatments can be introduced in a rolling fashion (or dropped, as appropriate). Thus, the trial is built to run perpetually as long as the disease and ideas to treat it exist.

In light of challenges conducting research relevant to pandemic infection uncovered by the 2009 H1N1 influenza experience, the REMAP-CAP trial was initially drafted with a pre-specified Pandemic Appendix to be activated in the event of an emergent pandemic. In February 2020, as the Coronavirus Disease 2019 (COVID-19) pandemic spread, the Pandemic Appendix to REMAP-CAP was activated and the trial labeled REMAP-COVID [4]. REMAP-COVID uses the same core design as REMAP-CAP, expands enrollment to include all hospitalized patients with clinically diagnosed or microbiologically confirmed COVID-19, adds COVID-19-specific treatment domains (Table 1), and enables the addition of new sites and regions. In this manuscript, we describe the implementation of the REMAP-COVID trial in the first US site, the UPMC health system, lessons learned, and recommendations for implementation at other sites.

**Table 1:** REMAP-COVID domains and arms. Additional domains can be found at www.remapcap.org

Domain	Arms		
**Antiviral**^**a**^	Hydroxychloroquine	Usual care	
**Corticosteroids**^**a**^	Low dose hydrocortisone	Moderate dose hydrocortisone	Usual care
**Immunoglobulin**^**b**^	Convalescent plasma	Usual care	Monoclonal antibodies
**Therapeutic Anticoagulation**^**b**^	Full dose anticoagulation (DVT/PE)	Thromboprophylaxis	
**Vitamin C**^**b**^	High dose vitamin C	Usual care	
**Immune Modulation 2 (IM2)**^**b,c**^	Eritoran^c^	Apremilast^c^	Usual care
**Platelet Inhibition**^**d**^	P2Y12 inhibitor	Aspirin	Usual care
**Statins**^**d**^	Simvastatin	Usual care	
**ACE2 RAS Modulation**^**d**^	ACE inhibitor	ARB^e^	Usual care
**Mechanical Ventilation**^**d**^	Protocolized mechanical ventilation	Usual care	

*DVT/PE* deep vein thrombosis/pulmonary embolus, *IND* Investigational New Drug, *IRB* Institutional Review Board, *ACE* angiotensin-converting enzyme inhibitor, *ARB* angiotensin receptor blocker

^a^Domain closed in the USA

^b^Domain actively enrolling in the USA, in partnership with the ATTACC and ACTIV4a (therapeutic anticoagulation), and LOVIT (Vitamin C) trials

^c^Enrolling as an IND domain under FDA oversight

^d^Protocol in development or pending activation

^e^A fourth arm of an ARB with a chemokine receptor-2 inhibitor may also be offered

To implement REMAP-COVID, we focused on six major areas (Table 2): engaging leadership, embedment into routine care processes and the electronic health record (EHR), remote consent and enrollment, regulatory compliance, modification of traditional trial management procedures, and alignment with other COVID-19 studies. We identified these areas by reviewing the major tasks we had done to launch the trial, proposing a finite list of conceptual areas that captured these tasks and would communicate to sites the work required to join the trial, and iteratively edited and completed the final list.

**Table 2:** Recommendations for implementation of the REMAP-COVID trial

Implementation area	Challenges	Recommendations
Leadership engagement	▪ Balancing dual imperatives of immediate patient care and research ▪ Diffuse interests	▪ Engage early ▪ Inclusive and transparent approach ▪ Align institutional and trial goals ▪ Understand and embrace bedside realities ▪ Share vision of REMAP-COVID as groundwork for learning health system development
Trial embedment	• Research procedures can interfere with daily care • Hospital committee workloads already high	• Embed trial into routine care processes • Integrate into existing institutional information dissemination, pharmacy, and telemedicine structures • Embed trial into electronic health record; can start trial using traditional methods
Remote consent and enrollment	• Technological challenges • Patient familiarity with technology	• Mock enrollments to test remote process • Engage bedside providers to assist patients as needed • Leverage institutional videoconferencing tools • Intermittent competency training of research personnel
Regulatory compliance and oversight	• Multiple parallel regulatory requirements	• Engage local institutional review board (IRB) to partner with the central IRB • Close contact with US Regional Coordinating Center at UPMC • Anticipatory management of new trial domains and arms
Modification of traditional trial management procedures	• Communication around frequent adaptive trial updates • COVID-19 travel restrictions limit in-person monitoring	• Identify and celebrate natural points of progress and contributions from local champions • Leverage electronic health record to facilitate protocol adherence, adverse event monitoring, screening and enrollment logs, and data collection and export • Start trial using traditional procedures • Virtual town hall meetings
Alignment with other COVID-19 studies	• Multiple studies for same patients	• Establish a centralized institutional process to optimize coordination and collaboration

*REMAP-COVID* Randomized Embedded Multifactorial Adaptive Platform for COVID-19

## Leadership engagement

The UPMC system is comprised of multiple community hospitals, regional tertiary referral centers, and one quaternary referral institution, all predominantly located in western and central Pennsylvania. For several years, the system has worked to develop a learning health system and previously launched a REMAP trial (https://clinicaltrials.gov/ct2/show/NCT03861767. https://clinicaltrials.gov/ct2/show/NCT03861767) [6] as part of an overall “Learning While Doing” program. This program seeks to accelerate development of a learning health system by encouraging synergy between the clinical research and clinical practice enterprises [7].

In January 2020, UPMC leadership decided to primarily keep patients with COVID-19 at the hospital to which they initially presented, with remote critical care support via telemedicine [8]. To ensure trial availability to all patients regardless of location, we engaged UPMC leadership to support implementation of REMAP-COVID across the system. We conducted meetings with administrative leaders, department chairs, informatics groups, the pharmacy and therapeutics committee, blood bank, and others to describe the vision of REMAP-COVID and propose specific implementation steps. Due to UPMC commitment to become a learning health system and to test novel therapies within trials, support was obtained, primarily in the form of access to existing infrastructure resources and institutional willingness to engage. In parallel, we engaged University of Pittsburgh leadership to collate and prioritize the multiple COVID-19 studies that were being proposed. The University of Pittsburgh and UPMC jointly designated the University of Pittsburgh Clinical and Translational Science Institute as the central hub for collecting information and providing resources related to COVID-19 studies, and investigators were asked to register their studies to optimize coordination.

## Trial embedment

### Embedment into routine care processes

A key REMAP design philosophy is for both the clinical care and clinical research enterprises to “lean in” towards one another [7], such that streamlined research processes become embedded within routine care processes, and informing future best practices becomes a part of daily care. To operationalize this philosophy, we identified opportunities to integrate REMAP-COVID into existing clinical care processes.

First, to promote trial awareness and enthusiasm, we worked with systemwide UPMC administrative groups to disseminate information via posting educational materials in the COVID-19 resources section of an internal UPMC website, adding trial announcements to COVID-19 communications from UPMC leadership, and identification of stakeholders at each hospital. We also presented the trial at Grand Rounds and other venues for virtual dissemination, to aid in reaching UPMC hospitals with limited prior research participation.

Second, we partnered with the pharmacy and therapeutics committee to set policy prioritizing use of experimental therapies within clinical trials, determine which REMAP-COVID investigational treatments were best suited for UPMC, and embed the trial into routine pharmacy operations. For example, the clinical pharmacies at each hospital dispense drugs with FDA Investigational New Drug (IND) exemptions, rather than an investigational pharmacy. To speed study launch, we began the trial only with interventions with IND exemptions. We educated pharmacists at the system level for each investigational treatment and emphasized medication order verification differences for COVID-19 versus non-COVID-19 indications.

Third, UPMC deployed a telemedicine program to facilitate local patient care and minimize COVID-19 transmission and provided tablet computers to help hospitalized patients communicate. We used these resources to aid remote connection with patients.

### Embedment into the electronic health record (EHR)

**Fig. 1:**
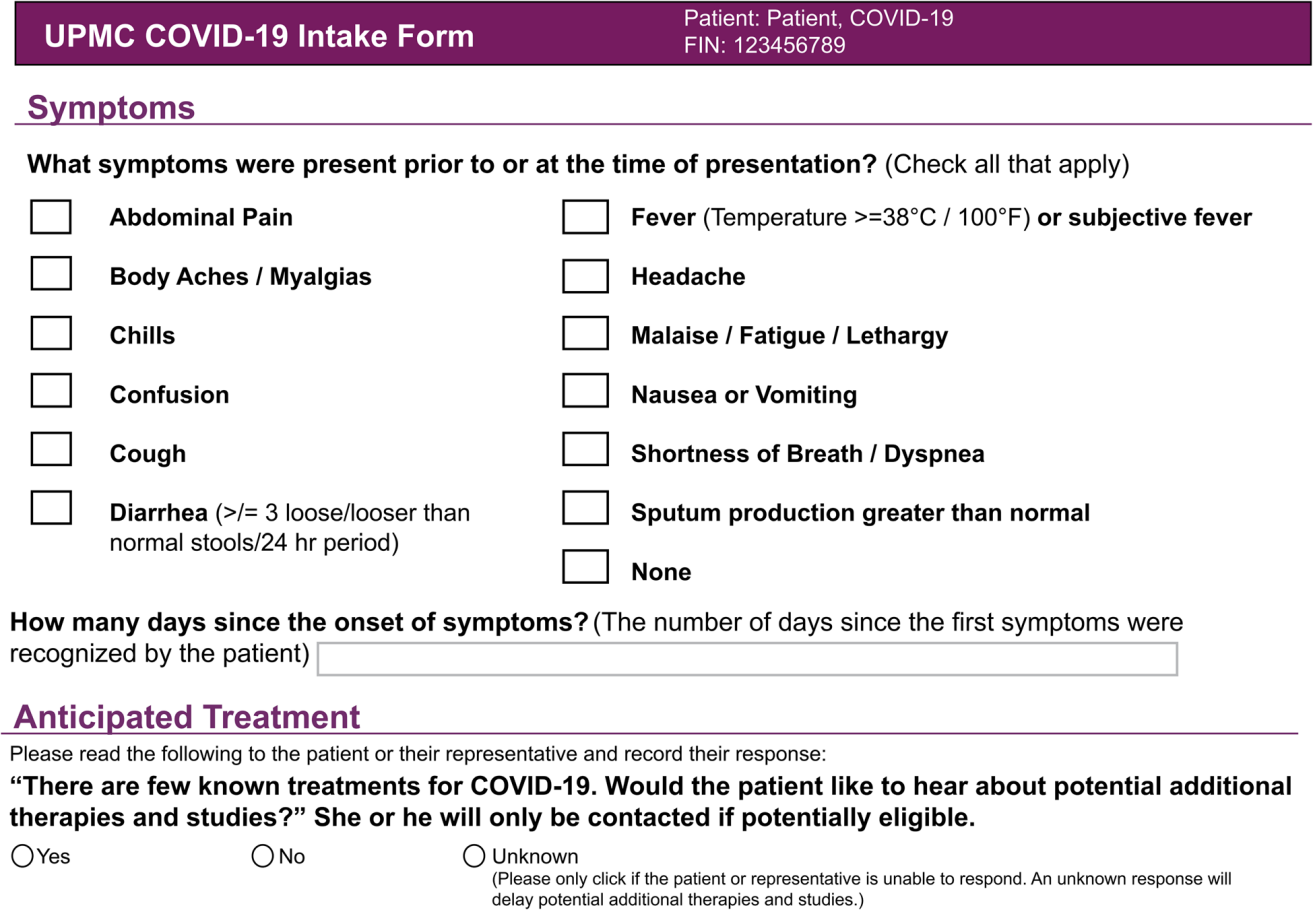
UPMC REMAP-COVID Intake Form. This form is embedded into a patient’s electronic health record, solicits basic clinical information, and requests providers to ask the patient or legally authorized representative if s/he is interested in potential additional therapies for COVID-19. The intake form represents the singular route of entry into the REMAP-COVID trial at UPMC

Embedment into the EHR facilitates low operational complexity at the bedside, despite complex internal trial machinery. We launched the trial at the 20 UPMC adult acute care hospitals that use Cerner (Cerner Corporation, North Kansas City, MO). To facilitate both clinical care and clinical research for COVID-19, we created a “one-stop shop” for operational efficiency—a COVID-19 tab in the provider facing interface of the patient’s electronic record. Physicians and advanced practice providers can use the tab to access treatment protocols and order sets, as well as complete a COVID-19 intake form (Fig. 1). The technology team programmed automated EHR alerts which prompt clinicians to complete the form when a SARS-CoV-2 test is ordered, a COVID positive isolation flag is entered, or a COVID order set is initiated, to capture basic data for surveillance reporting to public health authorities. Clinicians can suppress the form if they do not consider their patient to have active COVID-19.

The intake form also assists with identifying REMAP-COVID eligibility criteria and provides the only route for entry into the trial by requesting providers ask their patient or legally authorized representative (LAR) if they would be interested in hearing about potential additional therapies for COVID-19. An affirmative response represents assent to be approached about research, and intake form completion generates an automated email to research staff, which triggers the informed consent and enrollment process (described in the next section).

**Fig. 2:**
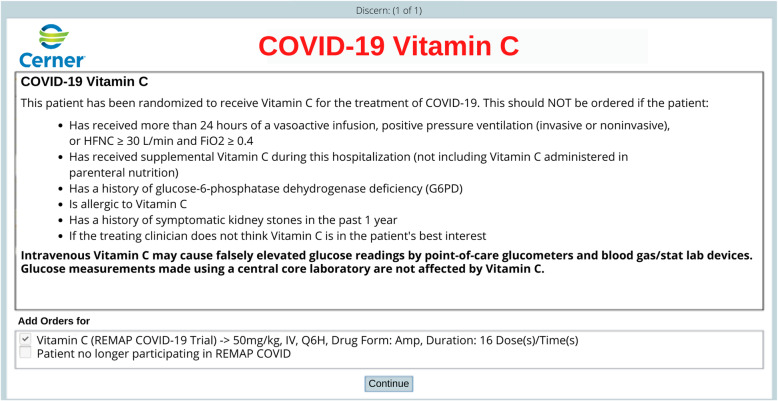
Example of an embedded order alert. This order alert displays the randomization status of the patient and asks the treating clinician to approve the order for the randomized investigational treatment unless deemed to be not in the patient’s best interest

After obtaining informed consent, randomization and in-trial notification processes are embedded within the EHR. Research staff complete a web-based application Enrollment Form, linked within Cerner, and hosted on a local server behind the health system firewall. The application references a response adaptive randomization table to generate treatment arm assignments for each domain (a trial “recipe”), which are then written by the application to a custom-built table within Cerner. Each unique trial recipe triggers a custom series of pop-up Cerner Discern alerts, which appear the next time after enrollment a provider enters a patient order. The alerts contain guidance for clinicians regarding eligibility criteria and protocol procedures. Where appropriate, the alerts pre-populate investigational treatment orders corresponding to the assigned trial recipe, which clinicians are requested to sign unless previously undetected exclusion criteria are present, or an investigational treatment is not thought to be in the patient’s best interest (Fig. 2). To further ensure in-trial notification, enrollment automatically triggers an icon in the banner bar that indicates the patient’s status as a trial participant, and a display of the assigned recipe on the COVID-19 tab in the patient’s record. Finally, research staff document an enrollment note in the EHR. If a patient declines participation or is ineligible, research staff document a trial note accordingly.

## Remote consent and enrollment

**Fig. 3:**
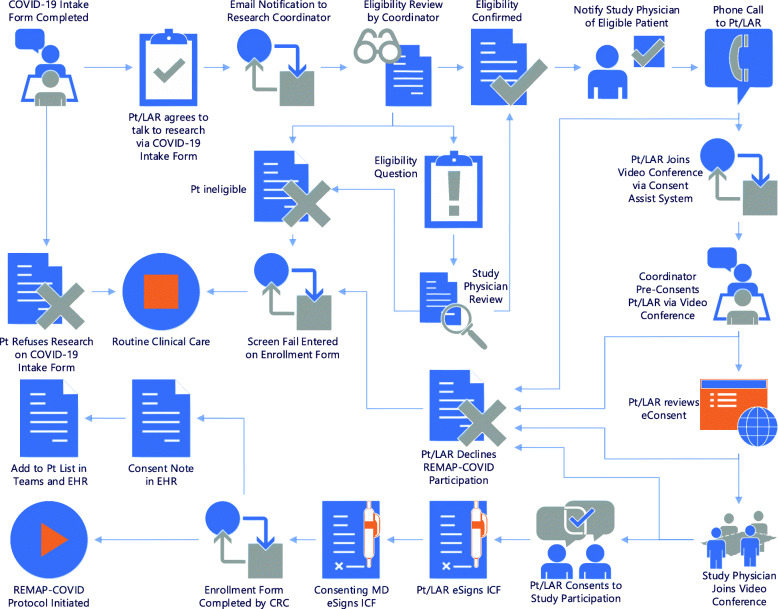
Remote consent and enrollment process. This process uses videoconference and document signing technologies to enable remote consent and enrollment

We developed a centralized remote consent and enrollment process (Fig. 3) applicable across all UPMC hospitals, to minimize COVID-19 transmission risk to research staff and preserve personal protective equipment.

Both the state of Pennsylvania and the University of Pittsburgh Institutional Review Board (IRB) require physician consent for clinical trials [9], with the University IRB requiring face-to-face consent, and a signed informed consent form is a clinical research standard [10]. Trial personnel execute four key steps in the remote consent process: (1) eligibility determination via chart review (with consultation of the treating clinician as needed); (2) a brief phone call to the patient’s personal or hospital room phone to introduce the trial; (3) a face-to-face consent discussion between the patient or legally authorized representative (hereafter, collectively referred to as “patient”), research staff, *and* physician investigator via secure video conference; and (4) signature capture via a software platform (e.g., DocuSign) that allows the patient to electronically sign their name using a smart device. We provide the software platform by texting or emailing a link to the patient’s smart device, investigators use the same platform to sign, and the final signed consent form is electronically sent to the patient. Bedside clinicians are engaged to assist with the process on an ad hoc basis, including providing patients who do not have a personal smart device a hospital phone or tablet, and assisting in the videoconferencing process if needed. Clinicians follow UPMC infection control procedures when assisting. On rare occasions, we have used paper consent, in which case a provider or investigator in personal protective equipment provides the paper forms and sends pictures of the signed forms to the research team.

A team of coordinators and multidisciplinary physician investigators trained in the protocol, informed consent, and Cerner currently screen and enroll 7 days a week, approximately 12 h a day. Once the informed consent signature is obtained, research staff then complete the web-based Enrollment Form, which triggers the randomization and alerts as described above.

## Regulatory compliance and oversight

UPMC and the University of Pittsburgh serve as both a REMAP-COVID site and the US Regional Coordinating Center. As most UPMC hospitals are under the purview of the University of Pittsburgh IRB, we obtained local approval to launch the trial. In parallel, we worked with the US sponsor, the Global Coalition for Adaptive Research (GCAR) to centralize protocol review at the Western Institutional Review Board (Puyallup, WA) for implementation across the USA. We presented each IRB the core protocol and appendices for each domain making the approval process modular and adaptable. Repurposed FDA-approved drugs are frequently granted an exemption from the Investigational New Drug (IND) application process, whereas experimental agents require IND approval. In partnership with GCAR, we submit documentation to the FDA for approval and adverse event monitoring where necessary. Trial registration at www.clinicaltrials.gov is updated with each newly activated domain (NCT02735707).

## Modification of traditional trial management procedures

We regularly update trial management procedures as the adaptive design necessitates frequent updates to our internal work environment [11], workflow [12], and data processes [13]. We work closely with UPMC information technology groups to add and drop study arms as the trial adapts, generate automated screening and eligibility logs, and create automated email alerts for all positive COVID-19 tests as a backup screening tool. To comply with COVID-19 travel restrictions, we remotely oversee trial execution at each hospital. To maintain trial awareness, we continuously reach out to hospitals across the system to promote the trial, identify local champions, and address questions. In addition, we recognize local champions for outstanding contributions and share trial updates through established UPMC information distribution mechanisms.

### Serious adverse event monitoring and protocol adherence

We use a combination of automated EHR detection alerts, traditional coordinator oversight via manual monitoring of enrolled patients, and input from clinical staff to monitor adverse events and protocol adherence. A REMAP goal is to rely on automated EHR detection alerts where possible as the trial progresses, and to optimize alerts by comparing them to manually adjudicated events. Medical monitoring is managed in a two-tiered system with a local investigator providing oversight of IND exempt interventions and a central medical monitor contracted by the sponsor for oversight of IND domains requiring FDA oversight. As the coordinating center for the US region of REMAP-CAP, essential documents are collected and filed with the UPMC program management team.

### Data collection and export

Trial-relevant data are continuously and automatically extracted from the Cerner database and curated into a dataset suitable for export to the international data coordinating center at Monash University in Australia. Data are stored in a MySQL Server database, processed using a scheduled combination of SQL statements and Python scripts, and formatted and exported for trial reporting. An unblinded investigator reviews initial exports in detail to ensure data accuracy. EHR data are reviewed for completeness and validity at the time of entry into the EHR, the time of extraction into a trial database, and following transformation into trial-relevant elements. Validation and process refinement are ongoing, to account for impurities in EHR data as well as frontline context of care and documentation practices that have changed due to the pandemic. A key challenge has been the need to rework both workflow and analytic models to keep pace with the adaptive nature of the trial and the evolution of the pandemic. To support adaptive randomization, relevant patient characteristics and outcomes are iteratively transferred to the international data coordinating center as a Health Insurance Portability and Accountability Act limited dataset using Globus (University of Chicago, Chicago, IL, USA) secure file transfer services. Post-hospital discharge outcomes are collected by a dedicated follow-up team via phone calls and query of national mortality databases. Secondary outcomes during hospitalization are captured from the EHR.

## Alignment with other COVID-19 studies

The COVID-19 pandemic sparked intense interest within academia and industry. To minimize competition for protocols, temper inconvenience to patients, regulate biospecimen volumes, and promote the “Learning While Doing” philosophy, UPMC and the University of Pittsburgh centralized all COVID-19 clinical trial recruitment through the REMAP infrastructure. As previously reported [4], the REMAP-COVID core protocol is broadly encompassing with basic eligibility criteria. As such, the REMAP-COVID core protocol and trial coordination team provide a gateway for entry into all COVID-19 clinical research, whereby trial personnel aid investigators of studies operating alongside REMAP in connecting with subjects. For example, consent for biospecimen collection and storage is coordinated with REMAP consent and allocation of specimens to individual investigators is centralized through a university assigned committee review; observational study data collection is integrated with REMAP EHR data extraction; and local investigator partnerships with industry are supported.

## Lessons learned and recommendations for implementation in other health systems

**Fig. 4:**
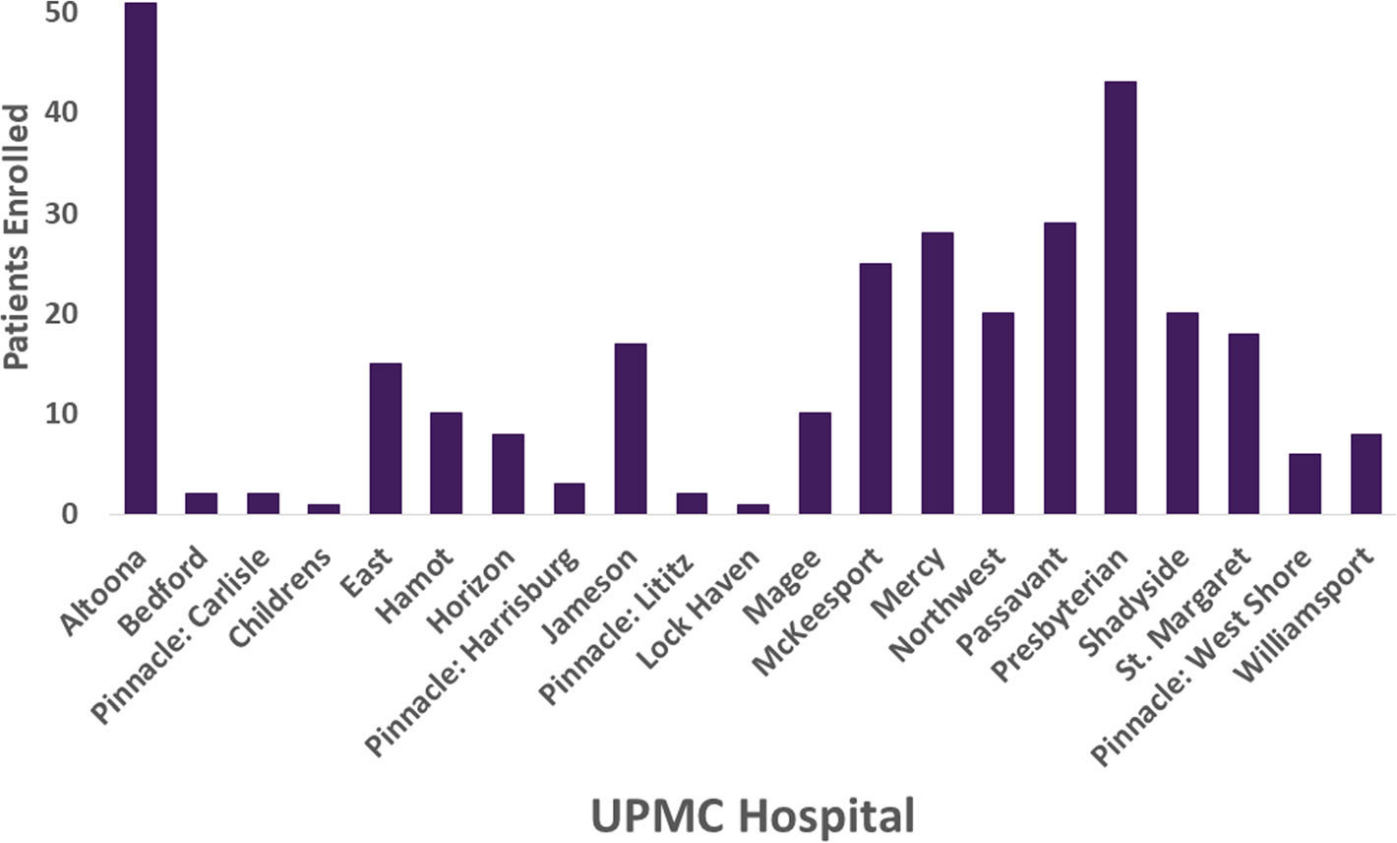
Current enrollment. As of Dec 14, 2020, there have been 319 patients enrolled among 2005 screened (16% enrollment) at 21 hospitals, after excluding those who were not candidates due to age < 18 years, not suspected to have COVID-19, unlikely to be admitted for more than 24 h due to either discharge or death, or having previously participated in REMAP COVID in the previous 90 days. The one patient enrolled at UPMC Children’s Hospital was older than 18 years of age but maintained longitudinal care with clinicians at Children’s

The pandemic challenged all REMAP-CAP sites to respond with innovation and efficiency. As a new site, we had limited preparation time before trial launch. We went live April 9, 2020, with two domains comprising three investigational treatment arms (hydroxychloroquine, moderate dose, and high dose hydrocortisone). We enrolled our first patient on April 12, added an immunoglobulin (convalescent plasma) domain on May 8, discontinued the initial two domains in response to emerging international data in mid-June [14–16], added Vitamin C and anticoagulation domains on July 23, and added an immunomodulation domain on October 21. The immunomodulation domain is under an IND and required coordination with the UPMC investigational pharmacy and additional steps to comply with FDA regulations. Additional domains are pending (Table 1). On September 21, we also went live with the seven adult acute care UPMC hospitals that use Epic (Epic Systems Corporation, Verona, WI), with an analogous EHR embedment process based on the one created for Cerner. As of December 14, 2020, we have enrolled 319 patients from 2005 screened (16% enrollment) at 21 hospitals (Fig. 4), after excluding those who were not candidates due to age < 18 years, not suspected to have COVID-19, unlikely to be admitted for more than 24 h due to either discharge or death, or having previously participated in REMAP-COVID in the previous 90 days. We are encouraged enrollment has occurred in community hospitals with limited prior research participation. Of the hospitals that have not yet enrolled a patient, most are low-volume, transfer patients to a regional center, or had ineligible patients.

We offer the following implementation recommendations, outlined in Table 2. As with the design transition of REMAP-CAP to the pandemic mode (REMAP-COVID), proactive and agile planning for implementation level is essential. All six implementation areas are necessary, with leadership engagement and, when applicable, alignment with other COVID-19 studies, the first priorities.

### Leadership engagement

We found an inclusive and transparent approach effective, while in retrospect we should have created and disseminated a preliminary organogram at the outset to rapidly communicate the complex trial structure. Although we had advantages of institutional desire and scale, in practice, implementing a learning health system (and REMAP-COVID) is achieved at the bedside and therefore local challenges with daily workflow and staffing require individualized attention. Similar conditions likely exist in other large health systems. Alignment of institutional and trial goals is imperative. Thus, simultaneously engaging leadership while keeping daily bedside realities in the forefront is essential. The optimal ways to engage leadership will vary by institution, as will unification needs, barriers, and solutions. Institution-level incentives for individual and group participation may increase bedside engagement and will also vary by institution. Participation in REMAP-COVID can help institutions lay the groundwork for future development as learning health systems. Smaller institutions may lack scale, but can likely move faster, and in the authors’ experience can match or exceed trial enrollment performance compared to larger institutions.

### Trial embedment

Embedment into routine care processes is essential as an implementation philosophy and design. We found partnering with existing institutional information dissemination, pharmacy, and telemedicine structures effective, and recommend this approach for efficient embedment, and as a means of engaging local leadership and front-line personnel.

While adding REMAP messages into existing information channels is relatively straightforward, constant and consistent re-education of frontline personnel is also required, particularly as investigational treatments are added or dropped. For drugs that require IND approval and are not available in clinical pharmacies, engagement of an investigational pharmacy is required, which may not be available in all hospitals. We have embedded the trial into the investigational pharmacies at the UPMC flagship academic hospitals and are expanding access to community hospitals using a combination of flagship hospital investigational pharmacy outreach and community hospital pharmacies.. We are also considering expanding intake form completion ability to pharmacists.

Many health systems have increased their telemedicine capabilities in response to the pandemic. This shift to telemedicine will likely be sustained, and opportunities to embed trials within existing telemedicine infrastructure should be sought. Our initial intent was to deploy telemedicine critical care nurses and physicians for informed consent and enrollment discussions. However, we abandoned this plan due to the complexity of ensuring all were trained in research ethics and protocol nuances, and uncertainty as to whether trial duties could coincide with telemedicine duties both operationally and ethically under current clinical research regulations. Nonetheless, it is our long-term goal to further embed trial operations into routine care processes and rely less on research personnel.

Embedment into the EHR is an optional feature, and traditional trial processes are utilized effectively in most regions of the world enrolling in REMAP-COVID. If feasible though, EHR embedment, while requiring significant upfront investment, renders trial operations more efficient than traditional processes and reduces both clinician and research staff burden, and positions institutions for future embedded research. In addition, EHR embedment allows enrollment at hospitals geographically distant from a centralized research team. Therefore, we recommend leveraging a system’s existing EHR to implement the trial if possible.

Customization of EHR-based workflow to support trial infrastructure, as has been accomplished within Cerner and Epic at UPMC, can conceivably be adapted to a range of other non-Cerner EHR systems. Most large health systems likely have the requisite information technology resources and expertise, while smaller systems may need to rely on traditional trial processes until standardized EHR solutions can be established. To this end, interactive web response system (IWRS) and electronic data capture (EDC) platforms have been established for REMAP-COVID to support trial deployment and software training resources are available from the coordinating center. Seamless to sites, data are collated, verified, and cleaned at the data coordinating center for analysis by the REMAP-CAP statistical analysis committee. In addition, data mapping across traditional IWRS/EDC systems and EHR-based approaches are handled at the coordinating center level.

### Remote consent and enrollment

We found remote consent and enrollment most effective when combined with engaged bedside care providers. Thus, as with implementation of any hospital-based project, stakeholder engagement cannot be overemphasized. Regulatory requirements for consent vary by state and institution. Videoconferences should be secure and electronic signature software should be compliant with part 11 of Title 21 of the Code of Federal Regulations [17]. If telephone consent is allowed, we recommend still having a video conference option should a patient or representative desire it. If coordinator consent is allowed, we recommend maintaining a physician investigator call pool, should a patient or representative wish to speak to a physician, and to support coordinators. Technological challenges including internet bandwidth for video connection, institutional firewalls, and patient smart device availability and familiarity require creative solutions and partnership with bedside providers. Thus, we recommend testing the remote consent and enrollment process prior to launch, including mock enrollments with a variety of individuals with varying familiarity with the protocol and with videoconferencing and electronic signature software. Similarly, intermittent competency training of research personnel to support real-time troubleshooting thought processes is recommended. Combining technological simplicity for the patient with regulatory compliance is essential.

### Regulatory compliance and oversight

We have streamlined the regulatory compliance and oversight processes to simplify onboarding of new sites in the US region in partnership with GCAR and the Western IRB. We recommend interested sites engage their local IRB to partner with the Western IRB for centralized ethics approval, and our group for protocol compliance, contract, and other key areas to facilitate rapid onboarding and activation of participant enrollment.

### Modification of traditional trial management procedures

Frequent adaptive trial updates and the lack of traditional milestones such as target enrollment numbers can be challenging for classically trained frequentist investigators and trial personnel. As recommended by other platform trials groups, we have found that identifying, pausing, and briefly celebrating natural points of progress and contributions from local champions aid morale [11]. Virtual town halls can efficiently augment outreach and trial awareness. Efficient adherence to trial conduct standards, including Good Clinical Practice (GCP) and data quality must be maintained. Protocol adherence, adverse event monitoring, screening and enrollment logs, and data collection and export for multiple hospitals can be facilitated by automated EHR extraction, combined with traditional manual oversight and validation. Sites can implement REMAP trial procedures using entirely traditional methods with research coordinator and investigator “boots on the ground” using IWRS-based randomization and electronic data capture, while developing more efficient EHR processes if resources are available.

### Alignment with other COVID-19 studies

Finally, implementation of the REMAP-COVID core protocol has enabled coordinated and collaborative clinical research focused on COVID-19 at UPMC. Other institutions have similarly centralized COVID-19 research [18, 19]. Establishing an integrated platform for clinical research will facilitate coordinated efforts not only during the pandemic but also into the future. For systems with minimal other COVID-19 research activity, such integration may be unnecessary, while for other large hospital systems integration is key.

### Future

We envision using the REMAP-COVID trial infrastructure to develop a learning healthcare system approach beyond COVID-19. The infrastructure can serve to implement best practices, facilitate quality improvement, and expedite clinical research. Participation of community hospitals is essential, expanding enrollment and generalizability beyond quaternary academic centers, as well as facilitating enrollment of vulnerable populations, who often disproportionately seek care locally rather than at large academic centers. Enrollment of patients by bedside clinicians as is done in the UK for the RECOVERY trial [20] is a long-term goal and will require enabling and incentivizing bedside clinicians to do so. REMAP-COVID provides an intermediate step to familiarize providers with research. Lastly, expansion of enrollment and data extraction protocols into non-Cerner EHR systems within UPMC can provide a framework for other US sites to facilitate EHR embedment.

In conclusion, implementation of the REMAP-COVID trial within a large US healthcare system is feasible and facilitated by multidisciplinary collaboration. This investment establishes important groundwork for future learning health system endeavors.

## Data Availability

Not applicable.

## Appendix

1. The UPMC REMAP-COVID Group

- *University of Pittsburgh Research Staff:* CRISMA Center—Kelsey Linstrum, Stephanie Montgomery, Kim Basile, Dara Stavor, Dylan Burbee, Amanda McNamara, Renee Wunderley, Nicole Bensen, Aaron Richardson; MACRO Center—Peter Adams, Tina Vita, Megan Buhay, Denise Scholl, Matthew Gilliam, James Winters, Kaleigh Doherty, Emily Berryman
- *UPMC Hospital Champions:* UPMC Altoona—Mehrdrad Ghaffari, UPMC East—Meghan Fitzpatrick; UPMC Jameson—Kavitha Bagavathy; UPMC Horizon—Kavitha Bagavathy; UPMC Mercy—Mahwish Hussain, Chenell Donadee; UPMC Williamsport—Emily Brant; UPMC McKeesport—Kayla Bryan-Morris, John Arnold and Bob Reynolds; UPMC Hamot—Gregory Beard; UPMC Presbyterian—Bryan McVerry, David Huang; UPMC Pinnacle—Janice Dunsavage, Salim Saiyed, Erik Hernandez, John Goldman, Cynthia Brown, Susan Comp, James Raczek, Jenny Lynne Morris, Jesus Vargas Jr., Daniel Weiss, Joseph W. Hensley, Erik Kochert, Chris Wnuk, Christopher Nemeth, Brent Mowery, Christina Hutchinson, Lauren Winters
- *UPMC Pharmacy:* Erin McCreary, Elise Martin, Ryan Bariola, Alex Viehman, Jessica Daley, Alyssa Lopus, Mark Schmidhofer, UPMC Directors of Pharmacy
- *UPMC ICU Service Center*: Rachel Sackrowitz, Chenell Donadee, Aimee Skrtich
- *UPMC Wolff Center*: Tami Minnier, Mary Kay Wisniewski, Katelyn Mayak
- *UPMC eRecord Team*: Richard Ambrosino, Sherbrina Keen, Sue Della Toffalo, Martha Stambaugh, Ken Trimmer, Reno Perri, Sherry Casali, Rebecca Medva, Brent Massar, Ashley Beyerl, Jason Burkey, Sheryl Keeler, Maryalyce Lowery, Lynne Oncea, Jason Daugherty, Chanthou Sevilla, Amy Woelke, Julie Dice, Lisa Weber, Jason Roth, Cindy Ferringer, Deborah Beer, Jessica Fesz, Lillian Carpio
- *Data Collection/Curation Team*: Salim Malakouti (Computer Science, University of Pittsburgh), Edvin Music and Dan Ricketts (CRISMA Center), Andrew King (Biomedical Informatics, University of Pittsburgh), Gilles Clermont (Critical Care Medicine), Robert Bart (UPMC Health Services Division)
- *UPMC Clinical Analytics*: Oscar Marroquin, Kevin Quinn, William Garrard, Kyle Kalchthaler
- *UPMC Office of Healthcare Innovation*: Derek Angus
- *Department of Emergency Medicine*: Alexandra Weissman, Donald Yealy, David Barton, Nadine Talia
- *Department of Critical Care Medicine*: David Huang, Florian Mayr, Andrew Schoenling, Mark Andreae, Varun Shetty, Emily Brant, Brian Malley, Chenell Donadee, Derek Angus, Christopher Horvat, Christopher Seymour, Timothy Girard, Gilles Clermont, Rachel Sackrowitz, Robert Bart
- *Division of Infectious Diseases*: Ghady Haidar
- *Division of Pulmonary, Allergy, and Critical Care Medicine*: Bryan McVerry, William Bain, Ian Barbash, Meghan Fitzpatrick, Christopher Franz, Georgios Kitsios, Kaveh Moghbeli, Brian Rosborough, Faraaz Shah, Tomeka Suber
- *Berry Consultants*: Roger Lewis, Michelle Detry, Anna McGlothlin, Christina Saunders, Mark Fitzgerald, Ashish Sanil, Scott Berry
- *Global Coalition for Adaptive Research (GCAR)*: Meredith Buxton, Brian Alexander, Tracie Roberts
- *REMAP-CAP International Steering Committee*: Steve Webb, Cameron Green, Colin McArthur

2. Protocol documents and the REMAP-CAP Investigators are listed at www.remapcap.org
