# Correction to: Implementation of the Randomized Embedded Multifactorial Adaptive Platform for COVID-19 (REMAP-COVID) trial in a US health system—lessons learned and recommendations

**DOI:** 10.1186/s13063-021-05109-8

**Published:** 2021-02-16

**Authors:** The UPMC REMAP-COVID Group, on behalf of the REMAP-CAP Investigators

**Affiliations:** 1 University of Pittsburgh, 606B Scaife Hall, Pittsburgh, PA 15213 USA; 2 UPMC Health System, Northwest 628, Pittsburgh, PA 15261 USA

**Correction to: Trials 22, 100 (2021)**

**https://doi.org/10.1186/s13063-020-04997-6**

Following publication of the original article [1], we were notified that a second corresponding author was missed.

The original article has been corrected to include Dr. McVerry’s email address.
